# Supplementary material for: Nitrate and Nitrite Metabolism in Aging Rats: A Comparative Study
Source: Nutrients. 2023 May 26;15(11):2490. doi: 10.3390/nu15112490 (PMC10255176; doi:10.3390/nu15112490)
Supplement: Supplementary file 1 [file nutrients-15-02490-s001.zip › nutrients-2378343-supplementary.pdf]

Table S1. Nitrate (**A**) and nitrite (**B**) levels in internal organs of young and old rats at baseline and after 5 days of nitrate treatment. TA: tibias anterior, EDL: extensor digitorum longus. Values are presented as average ± SD, n=4 for all organs.

A. Nitrate

**age**	**young**	**young**	**old**	**old**
**treatment**	**baseline**	**nitrate**	**baseline**	**nitrate**
**organ**	*(nmol/g)*	*(nmol/g)*	*(nmol/g)*	*(nmol/g)*
**liver**	11.1±3.5	15.7±6.3	12.5±7.2	27.4±10.5
**plasma**	15.1±3.9	58.8±30.8	7.6±1.6	65.8±33.9
**gluteus**	34.2±16.5	48.8±10.4	50.0±16.6	57.1±24.4
**eye**	8.5±0.4	15.5±2.0	1.0±0.1	3.3±1.6
**lacrimal glands**	15.5±4.5	26.2±9.1	11.1±5.3	62.6±15.5
**brain**	16.4±5.9	18.9±7.4	26.9±6.8	29.3±4.5
**salivary glands**	8.4±3.5	20.1±9.4	10.3±6.6	45.0±20.6
**heart**	22.9±3.6	26.0±10.1	14.7±6.0	23.3±9.4
**aorta**	10.5±2.3	24.6±10.2	8.9±1.5	34.9±10.7
**vena cava**	20.9±7.2	27.5±5.9	13.2±7.4	21.2±8.8
**TA**	28.7±21.6	61.6±26.7	49.8±5.9	81.4±25.4
**EDL**	30.3±19.1	62.6±12.7	68.0±16.8	69.2±23.7
**soleus**	38.2±26.4	59.4±34.2	67±23.0	72.8±10.3
**gastrocnemius**	24.8±8.4	39.9±17.0	40.3±12.5	43.7±9.9

B. Nitrite

age	young	young	old	old
treatment	baseline	nitrate	baseline	nitrate
**organ**	*(nmol/g)*	*(nmol/g)*	*(nmol/g)*	*(nmol/g)*
**liver**	0.36±0.11	0.44±0.14	0.24±0.07	0.40±0.11
**plasma**	0.24±0.08	0.59±0.15	0.27±0.03	0.46±0.08
**gluteus**	0.40±0.14	0.76±0.20	0.25±0.07	0.30±0.04
**eye**	0.25±0.01	0.49±0.11	0.18±0.06	0.24±0.02
**lacrimal glands**	0.62±0.11	0.78±0.29	0.31±0.18	0.37±0.34
**brain**	0.82±0.38	0.92±0.14	0.96±0.22	1.13±0.54
**salivary glands**	0.32±0.13	0.43±0.19	0.20±0.05	0.26±0.08
**heart**	0.32±0.09	0.41±0.10	0.37±0.05	0.69±0.20
**aorta**	1.02±0.43	2.31±0.86	0.53±0.11	1.48±0.58
**vena cava**	1.65±0.42	1.79±0.22	0.77±0.24	0.99±0.82
**TA**	0.34±0.10	0.81±0.29	0.39±0.03	0.35±0.05
**EDL**	0.31±0.06	0.55±0.32	0.46±0.11	0.54±0.16
**soleus**	0.40±0.05	0.81±0.48	0.46±0.03	0.54±0.08
**gastrocnemius**	0.31±0.07	0.56±0.22	0.38±0.08	0.39±0.04

Table S2. Relative changes of nitrate (**A**) and nitrite (**B**) content in organs and tissues of old and young rats. Changes are shown as fold increase after 5 days of nitrate treatment over baseline (left side) or as comparison of the same treatment across the age groups (right side). TA: tibias anterior, EDL: extensor digitorum longus.

A. Nitrate

	**nitrate/baseline**		**old/young**	
	**young rat**	**old rat**	**baseline**	**nitrate diet**
**liver**	1.41	2.19	1.13	1.75
**plasma**	3.89	8.66	0.50	1.12
**gluteus**	1.43	1.14	1.46	1.17
**eye**	1.82	3.30	0.12	0.21
**lacrimal glands**	1.69	5.64	0.72	2.39
**brain**	1.15	1.09	1.64	1.55
**salivary glands**	2.39	4.37	1.23	2.24
**heart**	1.14	1.59	0.64	0.90
**aorta**	2.34	3.92	0.85	1.42
**vena cava**	1.32	1.61	0.63	0.77

B. Nitrite

	nitrate/baseline		old/young	
	young rat	old rat	baseline	nitrate diet
**liver**	1.22	1.67	0.67	0.91
**plasma**	2.46	1.70	1.13	0.78
**gluteus**	1.90	1.20	0.63	0.39
**eye**	1.96	1.33	0.72	0.49
**lacrimal glands**	1.26	1.19	0.50	0.47
**brain**	1.12	1.18	1.17	1.23
**salivary glands**	1.34	1.30	0.63	0.60
**heart**	1.28	1.86	1.16	1.68
**aorta**	2.26	2.79	0.52	0.64
**vena cava**	1.08	1.29	0.47	0.55

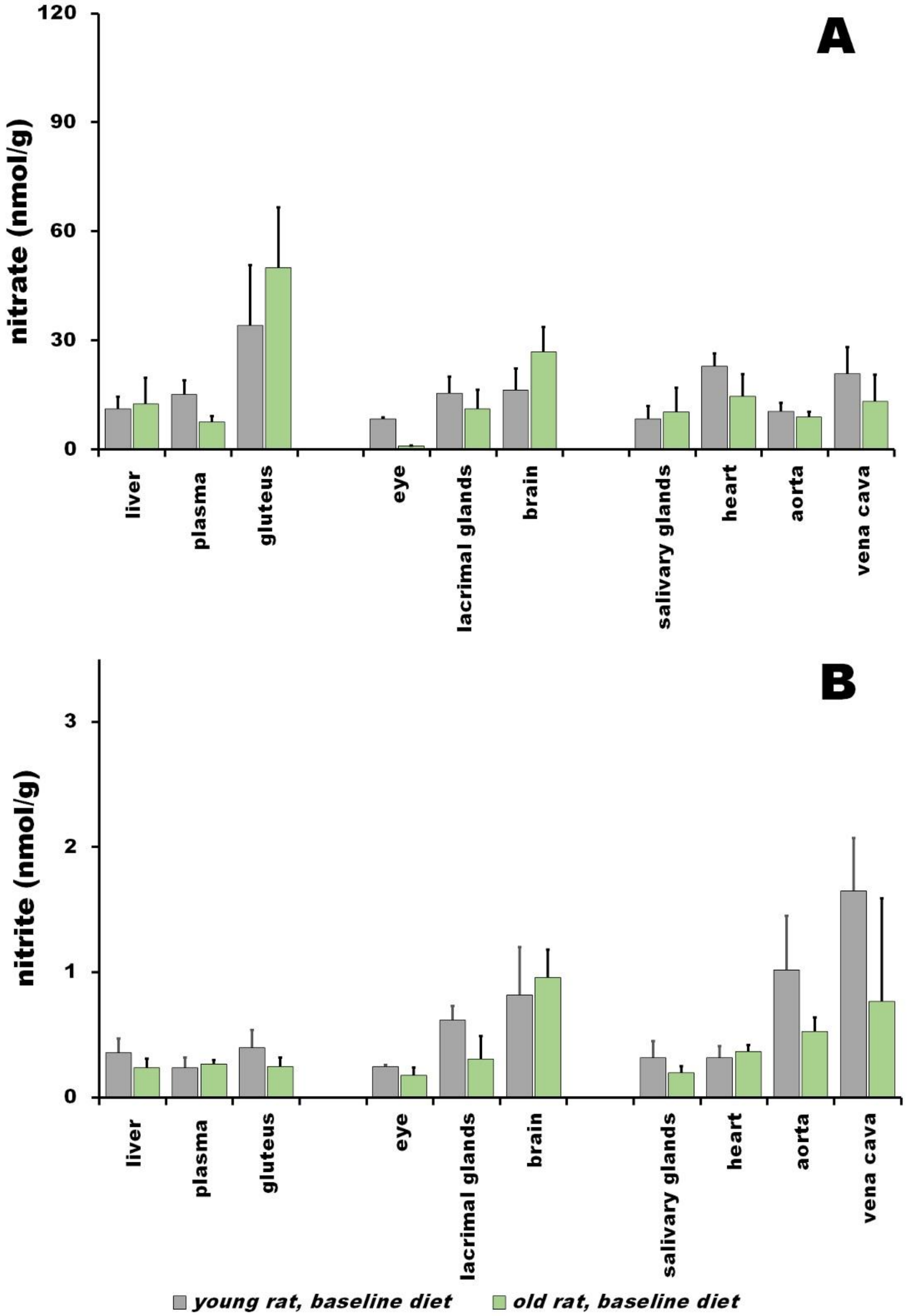

Figure S1. Nitrate (A) and nitrite (B) levels at baseline in young rats (light gray bar) and old rats (light green bar). Values are presented as average ± SD, n=4 for all organs.

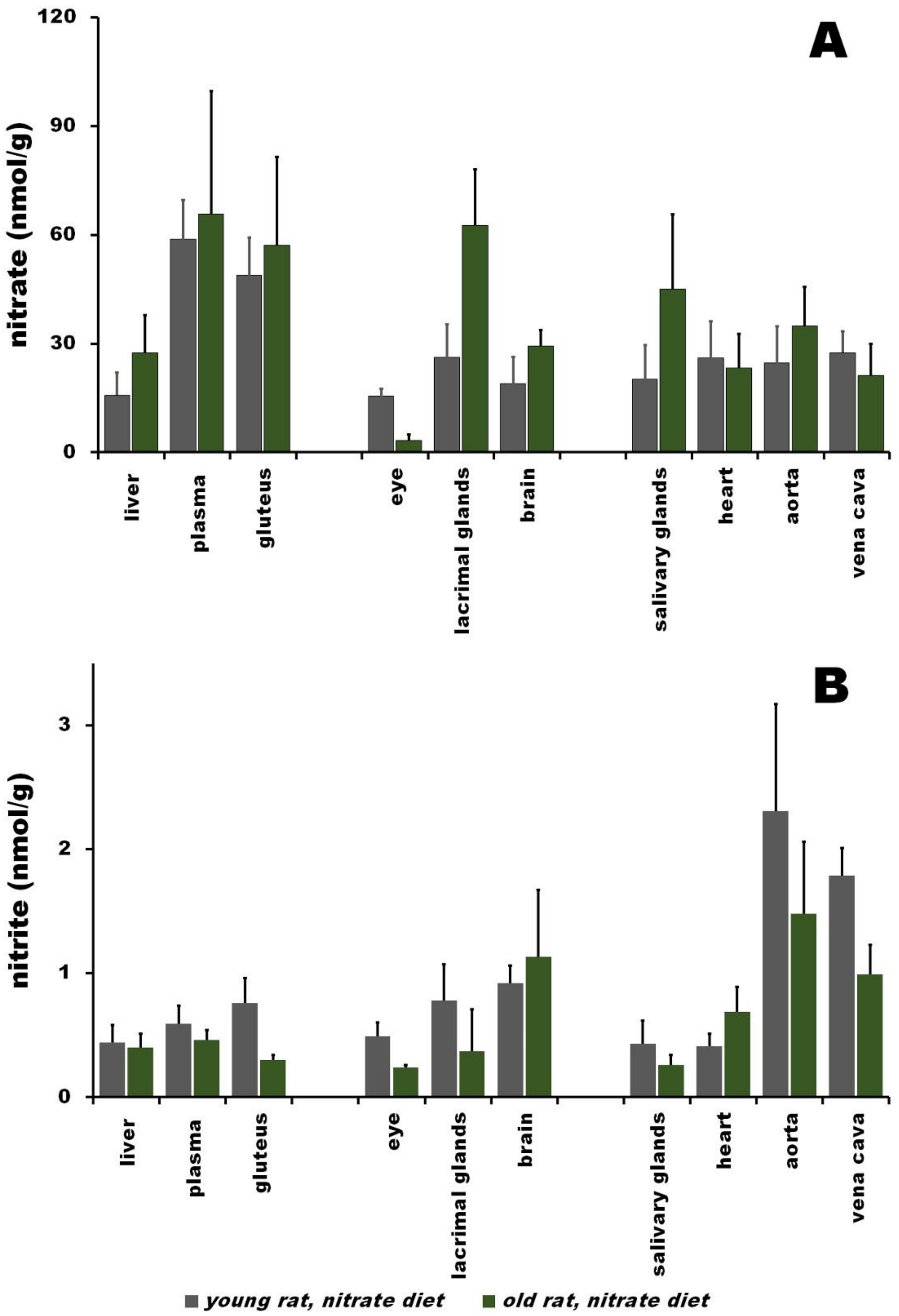

Figure S2. Nitrate (A) and nitrite (B) levels after 5 days of dietary nitrate supplementation in young rats (dark gray bar) and old rats (dark green bar). Values are presented as average ± SD, n=4 for all organs.

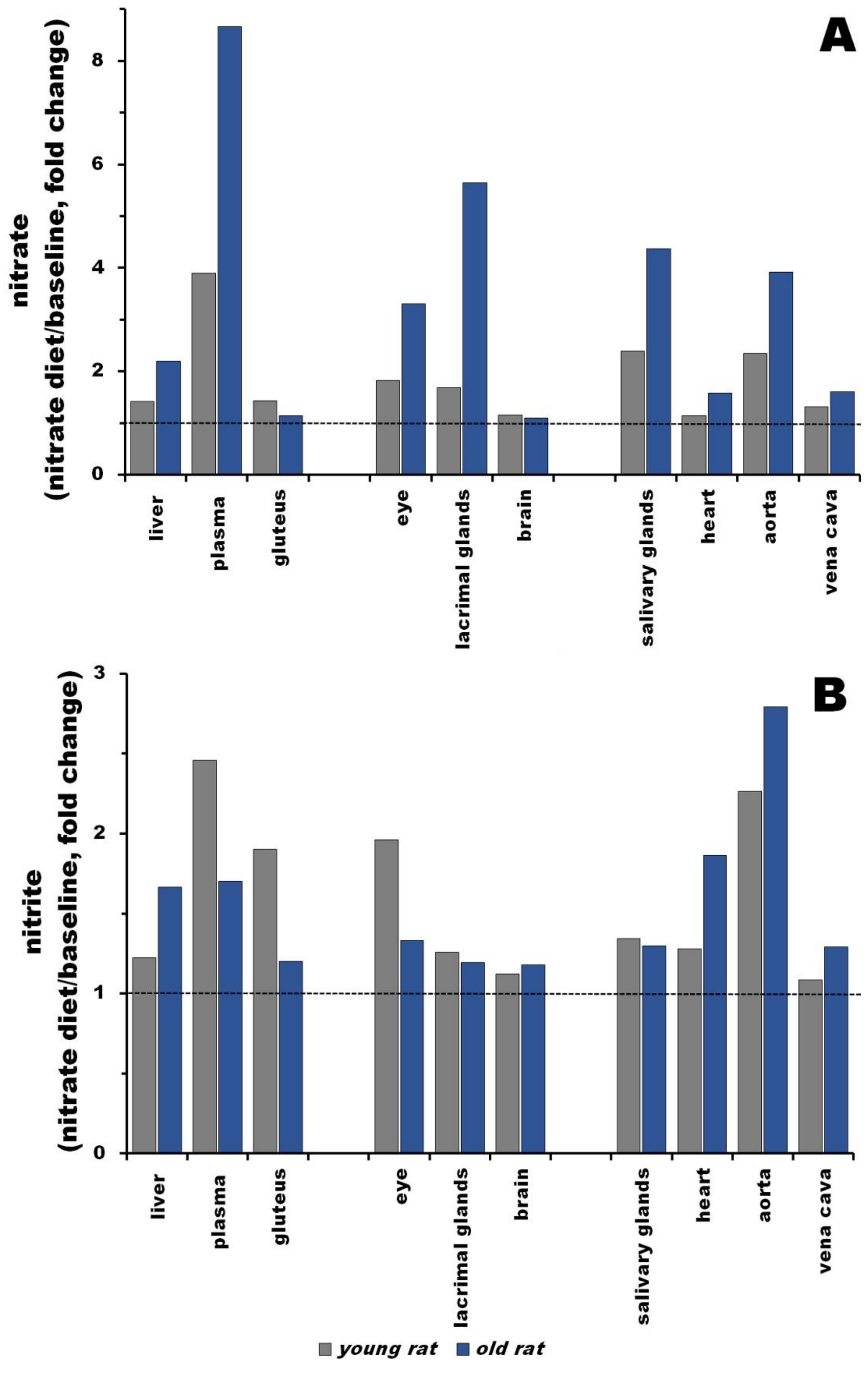

Figure S3. Ratio of nitrate (A) and nitrite (B) content in organs and tissues of young (gray bars) and old (blue bars) rats at baseline and after 5 days of nitrate supplementation. Ratio was calculated as concentration of nitrate (nitrite) after 5 days of nitrate

supplementation/nitrate (nitrite) at baseline. Ratio >1 reflects higher content of nitrate (nitrite) after dietary nitrate intake. Ratio <1 would reflect higher content of nitrate (nitrite) at baseline, which was not observed in any case.
